# Impact of Bicarbonate-β-Lactam Exposures on Methicillin-Resistant *Staphylococcus aureus* (MRSA) Gene Expression in Bicarbonate-β-Lactam-Responsive vs. Non-Responsive Strains

**DOI:** 10.3390/genes12111650

**Published:** 2021-10-20

**Authors:** Selvi C. Ersoy, Blake M. Hanson, Richard A. Proctor, Cesar A. Arias, Truc T. Tran, Henry F. Chambers, Arnold S. Bayer

**Affiliations:** 1 The Lundquist Institute, Torrance, CA 90502, USA; selvi.ersoy@lundquist.org; 2 Center for Infectious Diseases, School of Public Health, University of Texas Health Science Center, Houston, TX 77030, USA; Blake.Hanson@uth.tmc.edu (B.M.H.); CAArias@houstonmethodist.org (C.A.A.); 3 Center for Antimicrobial Resistance and Microbial Genomics, Division of Infectious Diseases, University of Texas Health Science Center at Houston McGovern Medical School, Houston, TX 77030, USA; tttran4@houstonmethodist.org; 4 School of Medicine and Public Health, University of Wisconsin, Madison, WI 53706-152, USA; rap@wisc.edu; 5 Houston Methodist Hospital, Houston, TX 77030, USA; 6 UCSF School of Medicine, San Francisco, CA 94143, USA; Henry.Chambers@ucsf.edu; 7 The Geffen School of Medicine at UCLA, Los Angeles, CA 90024, USA

**Keywords:** Methicillin-resistant *Staphylococcus aureus* (MRSA), β-lactam susceptibility, sodium bicarbonate (NaHCO_3_), RNA-sequencing (RNA-seq)

## Abstract

Methicillin-resistant *Staphylococcus aureus* (MRSA) infections represent a difficult clinical treatment issue. Recently, a novel phenotype was discovered amongst selected MRSA which exhibited enhanced β-lactam susceptibility in vitro in the presence of NaHCO_3_ (termed ‘NaHCO_3_-responsiveness’). This increased β-lactam susceptibility phenotype has been verified in both ex vivo and in vivo models. Mechanistic studies to-date have implicated NaHCO_3_-mediated repression of genes involved in the production, as well as maturation, of the alternative penicillin-binding protein (PBP) 2a, a necessary component of MRSA β-lactam resistance. Herein, we utilized RNA-sequencing (RNA-seq) to identify genes that were differentially expressed in NaHCO_3_-responsive (MRSA 11/11) vs. non-responsive (COL) strains, in the presence vs. absence of NaHCO_3_-β-lactam co-exposures. These investigations revealed that NaHCO_3_ selectively repressed the expression of a cadre of genes in strain 11/11 known to be a part of the *sigB*-*sarA*-*agr* regulon, as well as a number of genes involved in the anchoring of cell wall proteins in MRSA. Moreover, several genes related to autolysis, cell division, and cell wall biosynthesis/remodeling, were also selectively impacted by NaHCO_3_-OXA exposure in the NaHCO_3_-responsive strain MRSA 11/11. These outcomes provide an important framework for further studies to mechanistically verify the functional relevance of these genetic perturbations to the NaHCO_3_-responsiveness phenotype in MRSA.

## 1. Introduction

The rise of antibiotic-resistant bacteria has increasingly threatened healthcare initiatives worldwide [1,2,3,4]. In particular, methicillin-resistant *Staphylococcus aureus* (MRSA) is a major clinical pathogen, causing a wide variety of severe infections, with relatively limited treatment options, poor patient outcomes, and increased healthcare-associated costs [5,6,7,8]. MRSA strains are traditionally considered as “resistant” to nearly all β-lactams (the treatment of choice for methicillin-susceptible *S. aureus* [MSSA]) [9,10]. Standard treatment options for MRSA, such as vancomycin or daptomycin, tend to be less effective, less well tolerated (in terms of side effects and toxicities) and more expensive than standard-of-care β-lactams as used for MSSA infections [11,12,13,14].

Recently, a novel β-lactam susceptibility phenotype was discovered among MRSA strains, termed ‘bicarbonate [NaHCO_3_]-responsiveness’, wherein certain MRSA strains become sensitized to β-lactam antibiotics in vitro in the presence of NaHCO_3_ [15,16]. This phenotype was relatively widespread among a small collection (n = 58) of US bloodstream isolates (ranging from 33–75% for oxacillin and cefazolin, respectively [16]). Importantly, the translatability of this phenotype has been verified both ex vivo (in simulated endocarditis vegetations [SEV] models) and in vivo (in experimental infective endocarditis models) [15,17]. To-date, the potential mechanisms of this phenotype have focused on the impact of NaHCO_3_ on *mecA* expression/PBP2a protein production, as well as upon genes associated with PBP2a maturation and functionality [15,18]. However, these mechanistic studies have predominantly centered on targeted investigations of well-characterized genes already known for their involvement in β-lactam resistance in MRSA, rather than a more unbiased and global consideration of genes that are selectively impacted among NaHCO_3_-responsive vs. non-responsive MRSA strains.

In the present investigation, we have utilized RNA-sequencing (RNA-seq) methods to identify a broader repertoire of genes likely involved in the NaHCO_3_-responsive vs. non-responsive phenotypes. We concentrated on identifying genes which were selectively expressed during NaHCO_3_-β-lactam co-exposures, comparing a prototype NaHCO_3_-responsive strain (MRSA 11/11) to a prototype non-responsive strain (COL) [15,17,18]. These RNA-seq analyses revealed a number of differentially and selectively expressed genes in the NaHCO_3_-responsive strain, with several noteworthy gene cadres, including: (i) genes within the *sigB*-*sarA*-*agr* regulatory axis; (ii) cell wall-anchored/peptidoglycan-associated genes; and (iii) genes involved in either global or divisome–specific autolysis.

## 2. Materials and Methods

### 2.1. Bacterial Strains and Growth Conditions

MRSA strains MRSA 11/11 (USA300) and COL (USA100) were utilized as representative NaHCO_3_-responsive and -non-responsive strains, respectively [15]. These two prototype strains have been used in a number of our prior investigations related to NaHCO_3_-responsiveness in vitro, ex vivo and in vivo [15,17,18]. These strains have the following *mecA*, *agr*, *spa* and CC types: SCC*mec* IV, *agr* I, t008, CC8 (MRSA 11/11); and SCC*mec* I, *agr* I, t008, CC8 (COL).

Strains were stored at −80 °C until thawed for use, and isolated on tryptic soy agar (TSA). Strains were grown in cation-adjusted Mueller Hinton Broth (CA-MHB; Difco) + 100 mM Tris (pH 7.3 ± 0.1) with or without 44 mM NaHCO_3_ or CA-MHB + 100 mM Tris (pH 7.3 ± 0.1) + 2% NaCl + ½ x the minimum inhibitory concentration (MIC) of oxacillin (OXA), with or without 44 mM NaHCO_3_ to generate RNA to compare the impact of NaHCO_3_ alone or the impact of NaHCO_3_ + OXA, respectively, by RNA-seq. OXA concentrations for MRSA 11/11 were 16 µg/mL (CA-MHB Tris) and 0.25 µg/mL (CA-MHB Tris + 44 mM NaHCO_3_) (representing ½ the MIC as determined for this strain by broth microdilution in media without and with NaHCO_3_, respectively [15]); OXA concentrations for COL were 256 µg/mL for both distinct growth conditions. We have used the above NaHCO_3_ and OXA concentrations in our prior studies [15,16,17,18,19]. Although this NaHCO_3_ concentration (44 mM) is above human blood levels, it replicates tissue levels [20], and maximizes the NaHCO_3_-responsiveness phenotype [15,19].

### 2.2. RNA Isolation, Library Construction, Sequencing and Analysis

To obtain RNA, strains were grown in CA-MHB 100 mM Tris ± 44 mM NaHCO_3_ overnight (O/N), then diluted 1:50 into 25 mL of either the same O/N growth medium or O/N growth medium supplemented with 2% NaCl and ½ x OXA MIC concentrations (as indicated above). Cultures were then grown for 4 h at 37 °C with aeration (to reach an OD_600_ ~0.5), and total RNA was harvested by mechanical disruption by FastPrep disruption (MP Biomedicals, Irvine, CA, USA), followed by column isolation (Qiagen, Germantown, MD, USA), and treatment with Turbo DNAse (Invitrogen, Waltham, MA, USA) to remove DNA from sample [15]. Ribosomal RNA was depleted using the Ribo-Zero Magnetic Bead rRNA Removal Kit (Illumina, San Diego, CA, USA) and prepared for sequencing using the Illumina Stranded Total RNA Prep Kit (Illumina, San Diego, CA, USA). Extracted RNA quality was assessed with an Agilent TapeStation 4200 (Agilent, Santa Clara, CA, USA) using an RNA ScreenTape, assuring an RNA Integrity Number (RIN score) above 6 before moving into library preparation. Following cDNA conversion, fragment sizes were assessed using an Agilent TapeStation 4200 and a High Sensitivity DNA ScreenTape, and DNA concentration was assessed using a Qubit (Thermo Fisher Scientific, Waltham, MA, USA) and dsDNA HS Assay kit. Sequencing libraries were pooled at equimolar concentrations and sequenced on an Illumina HiSeq 4000 (Illumina, San Diego, CA, USA) using 2 × 150 paired end reads. Sequencing adapters and low-complexity reads were removed using Trimmomatic v0.38 [21], and aligned to the ASM1346v1 and ASM1204v1 RefSeq genomes with Bowtie2 v2.3.4.2 for MRSA 11/11 and COL, respectively [22]. Transcripts were assembled using Cufflinks v2.2.1, and CummeRbund v2.32.0 [23]. Two biological replicates were assessed in technical duplicate for each strain and indicated growth condition. Expression data for these samples containing NaHCO_3_ are currently available in the NCBI Sequence Read Archive (SRA) database under the accession numbers SAMN21542115 (MRSA 11/11 CA-MHB Tris + NaHCO_3_), SAMN21542148 (MRSA 11/11 CA-MHB Tris + OXA + NaHCO_3_), SAMN21542116 (COL CA-MHB Tris + NaHCO_3_), and SAMN21542147 (COL CA-MHB Tris + OXA + NaHCO_3_). Codification of the accession numbers for sequence read data-basing for samples that do not contain NaHCO_3_ (MRSA 11/11 CA-MHB Tris, MRSA 11/11 CA-MHB Tris + OXA, COL CA-MHB Tris, COL CA-MHB Tris + OXA), are currently in-progress and will be made available by contacting the lead Author (S.C.E.), at the following email address (selvi.ersoy@lundquist.org). “Differentially and selectively expressed genes” in our study represented those genes which exhibited a significant level of differential expression in the presence of NaHCO_3_±OXA exposure compared to media without NaHCO_3_ for the responsive strain, MRSA 11/11 vs. the non-responsive COL strain (≥ 2-fold). Thus, this definition explicitly eliminated genes which were differentially and concordantly expressed under these conditions in MRSA 11/11 as well as in COL. For selected analyses, we catalogued genes which were differentially expressed by ≥ 5-fold.

Kyoto Encyclopedia of Genes and Genomes (KEGG) metabolic pathways and annotations for differentially abundant genes were mapped using KOBAS v3.0 [24].

### 2.3. qRT-PCR Validation of Select Genes

Total RNA was isolated from MRSA 11/11 and COL log phase cells (OD_600_ = 0.5) grown in CA-MHB 100 mM Tris + 2% NaCl + ½ × MIC OXA ± 44 mM NaHCO_3_ as previously described [15]. Briefly, RNA was extracted by column isolation (Qiagen, Germantown, MD, USA) following mechanical disruption by FastPrep (MP Biomedicals, Irvine, CA, USA). Total RNA was treated with Turbo DNAse (Invitrogen, Waltham, MA, USA) and reverse transcribed by random hexamers to generate a cDNA library with SuperScript IV (Invitrogen, Waltham, MA, USA).

Following RNA-seq analyses, we selected five genes from the overall repertoire of differentially expressed genes for qRT-PCR validations, representing broad categories of bacterial factors, including: i) peptidoglycan synthesis (*pbp2* and *ddh*); ii) autolysis (*atl* and *sceD*); and iii) virulence (*fnbA*). The primers listed in Table S1 were used to amplify each gene-of-interest to determine their relative expressions. The gene *gyrB* was used as a housekeeping gene to normalize transcript abundance. The qRT-PCR was carried out on a StepOne thermocylcer (ThermoFisher, Waltham, MA, USA) and analyzed with StepOne Software. Relative gene expression was calculated using the 2^−ΔΔC^_T_ method from two independent biological replicates performed in triplicate on at least two separate runs for each strain/condition. Relative gene expression in CA-MHB 100 mM Tris+2% NaCl+½ × MIC OXA+44 mM NaHCO_3_ was normalized to CA-MHB 100 mM Tris+2% NaCl+½ × MIC OXA for each gene, with expression in the latter condition set equal to 1.0. statistical significances related to relative expression profiles were determined by a Student’s *t*-test, with a *p* value of ≤0.05 considered ‘significant’.

## 3. Results and Discussion

### 3.1. Differentially Expressed Genes by RNA-seq in NaHCO_3_-responsive vs. Non-responsive Strains

**Figure 1:**
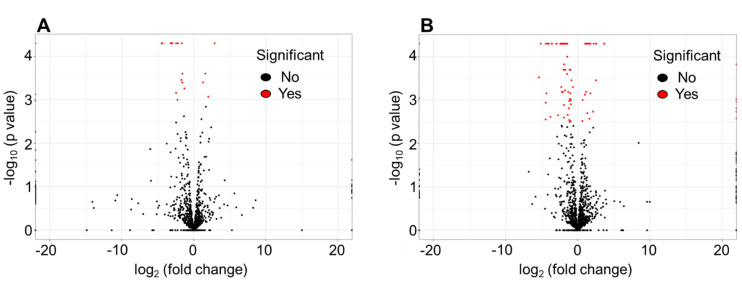
Volcano plots of the differential expression of genes in the presence of: (**A**) NaHCO_3_ exposure alone; or (**B**) NaHCO_3_ +½xMIC OXA co-exposures in NaHCO_3_-responsive strain MRSA 11/11. NaHCO_3_ alone led to an observed 21 differentially expressed transcripts. NaHCO_3_ and OXA co-exposures together yielded 84 differentially expressed transcripts. These numbers represent the total number of transcripts identified, featuring genes of both known and unknown functions.

RNA-seq analysis was carried out on two prototypical MRSA strains, MRSA 11/11 (NaHCO_3_-reponsive) and COL (NaHCO_3_-non-responsive), for two distinct comparison groups: Group 1—CA-MHB Tris vs. CA-MHB Tris + NaHCO_3_; and Group 2—CA-MHB Tris + 2% NaCl + OXA vs. CA-MHB Tris + NaHCO_3_ + 2% NaCl + OXA. RNA-seq analysis of the prototypical NaHCO_3_-responsive strain MRSA 11/11 revealed that exposure to NaHCO_3_ alone (Group 1) led to the differential change in abundance (≥ 2-fold change) of 21 transcripts (Figure 1A); in contrast, NaHCO_3_-OXA combination (Group 2) yielded a differential change in abundance of 84 transcripts (Figure 1B). Following initial analysis of differentially abundant transcripts for Groups 1 and 2 in MRSA 11/11 and COL, those genes whose expression was altered in the same direction for both MRSA 11/11 and COL in each group were subtracted from subsequent analyses, as presented in Table 1. 

NaHCO_3_ exposure, in the presence and absence of β-lactam co-exposure, impacted the expression of genes across a range of functional groups, with a large proportion relating to either cell wall synthesis, cell membrane transporters, or cellular metabolism (Table 1). Intriguingly, across most functional categories, NaHCO_3_ exposure resulted in significant repression of gene expression, particularly for those genes relating to virulence, cell wall biosynthesis, and membrane transporters (Table 1, Table S2A–C). Of the genes that were upregulated by NaHCO_3_ exposure, these tended to be related to cellular metabolism, protein translation, and/or stress response, indicating that NaHCO_3_ may mirror the exposure of the responsive MRSA strain to an ‘inhospitable’ host environment.

Oxidative and membrane stress have been implicated in the formation of persister cells [25], which make up approximately 1% of the total cell population; such cells have increased antibiotic tolerance and reduced metabolism [26,27,28]. The RNA-seq data above indicate that, while NaHCO_3_ may be stimulating a stress response in NaHCO_3_-responsive MRSA, NaHCO_3_ also evoked increased expression of many metabolic genes as compared to cells grown in the absence of NaHCO_3_. This latter finding (i.e., enhanced metabolic activity) would not support the notion of NaHCO_3_-mediated entry of responsive MRSA cells into a persister state (Table 1, Table S2A–C).

**Figure 2:**
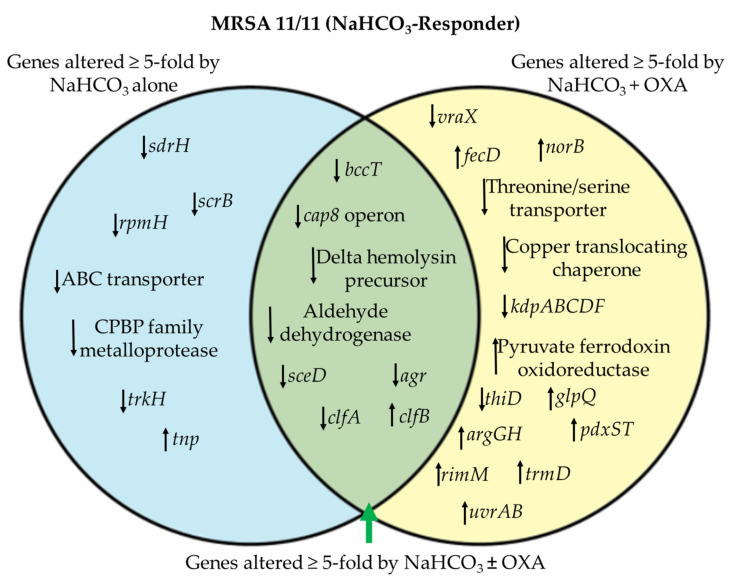
Venn diagram of genes altered ≥ 5-fold by NaHCO_3_ exposure without (left) or with (right) OXA exposure specifically in NaHCO_3_-responsive strain MRSA 11/11. Arrows indicate whether expression was decreased (downward arrow) or increased (upward arrow) by NaHCO_3_ exposure. OXA exposure is equivalent to ½ × MIC of OXA in CA-MHB Tris + 2% NaCl ± NaHCO_3_.

We then interrogated only those differentially abundant transcriptional units that were specifically altered in MRSA 11/11, and for which a specific function is known and expression was altered by ≥ 5-fold. NaHCO_3_ alone significantly impacted the expression at this magnitude of only a relatively small number of functionally-identifiable genes/transcriptional units (n = 7) as compared to a larger number of genes/transcriptional units differentially impacted by NaHCO_3_+OXA (n = 14) in the NaHCO_3_-responsive strain MRSA 11/11 (Figure 2). Additionally, eight transcriptional units were differentially expressed in both growth conditions (NaHCO_3_ alone and NaHCO_3_+OXA) (Figure 2).

Of particular interest, the autolysin *sceD* and the global virulence regulator *agr* were both substantially repressed (≥ 5-fold change in expression) by NaHCO_3_, with or without β-lactam co-exposure (Figure 2, Table 2). The *agr* locus is a major virulence regulator in *S. aureus* [29,30,31], although its role in antibiotic resistance in vivo remains controversial [30,32]. Previously, we observed by qRT-PCR that NaHCO_3_ is capable of repressing the global virulence regulons *sarA* and *sigB* (both *agr*-regulated) [15,18]. These RNA-seq data corroborate the downstream effects of *sarA* and *sigB* repression, as observed by the repression of *agr* and several other genes known to be part of the *sigB-sarA*-*agr* regulon, including *fnbA*, *fnbB*, *cap8*, *clfA*, and *clpL* (Table 2, Table 3) [33,34,35,36]. Of note, one of the few genes upregulated by NaHCO_3_ exposure in MRSA 11/11 was *clfB*, a cell wall-anchored (CWA) protein whose function is linked to nasal colonization and virulence in skin and soft tissue infections [37]. Additionally, NaHCO_3_ repressed expression of *sdrH*, another CWA gene whose repression is associated with decreased biofilm formation [38,39], an impact that corresponds to the observed decrease in biofilm formation during NaHCO_3_ exposure in MRSA 11/11 [40]. Taken together, these data indicate a broad impact of NaHCO_3_ (especially in combination with OXA) on genes associated with cell surface modifications, as well as cell wall biosynthesis or remodeling.

It should be underscored that NaHCO_3_ did increase the expression of several genes in the non-responsive strain, COL, whose expressions were down-regulated in MRSA 11/11, including the *cap8* operon, the virulence-associated gene *vraX*, and the potassium transport operon *kdpABCDF* (Table 3). Despite these observed differences in such putative virulence gene expressions between COL and MRSA 11/11, we have not observed any differences in the intrinsic virulence of these two strains in vivo [15]. Further, a large cadre of genes was specifically impacted by NaHCO_3_, with or without OXA exposure in strain COL, but not in MRSA 11/11. However, the analytics required to determine the role of these specifically and differentially impacted genes in generating the non-responsive MRSA phenotype is beyond the scope of this paper.

Of primary interest in regard to NaHCO_3_-mediated susceptibility to β-lactams was its impact on the expression of genes related to cell wall synthesis. NaHCO_3_ (with or without OXA co-exposure) repressed the expression of two key autolysins, *atl* and *sceD*; in addition, the expressions of several other key genes involved in cell wall synthesis were repressed by NaHCO_3_-OXA co-exposures (*isaA*, *fmtA*, *ddh*, *pbp2*) (Table 2). These latter six genes encompass those involved in the formation of nascent (new) peptidoglycan and cell wall restructuring (*pbp2*, *ddh*, *fmtA*) [41,42,43,44], as well as peptidoglycan hydrolases, involved in cell wall turnover and division (*atl*, *sceD*, *isaA*) [45,46,47]. Previous studies have demonstrated that the deletion or inactivation of *isaA* and *fmtA* results in increased susceptibility to β-lactams, an event independent of impacts on the expression of PBP2a [45,48]. Furthermore, the regulation of *sceD* and *isaA* transcription is linked, since deletion in *isaA* results in enhanced expression of *sceD* [45,46]. We observed a modest decrease in *isaA* transcription in the presence of NaHCO_3_-OXA, but a substantial decrease in *sceD* transcription in the presence of NaHCO_3_ (with or without OXA). Although previous reports did not find that the deletion of *sceD* alone resulted in increased β-lactam susceptibility [45], it is possible that within the context of the gene expression profile stimulated by NaHCO_3_, reduced *sceD* expression may have a larger impact on β-lactam susceptibility than previously understood.

### 3.2. qRT-PCR Validation

**Figure 3:**
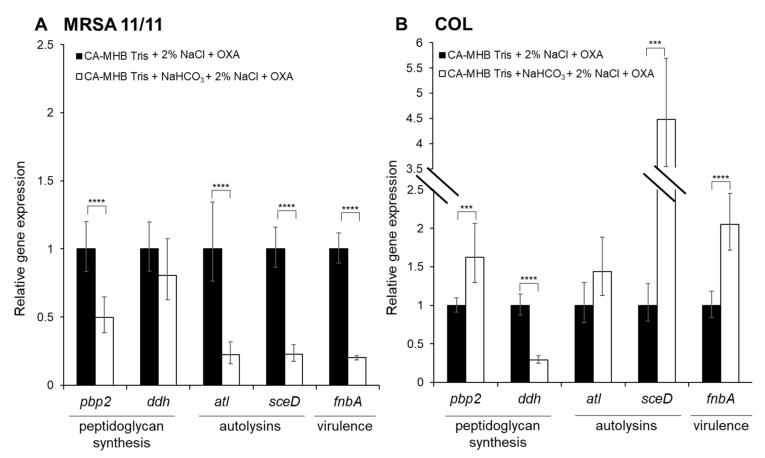
qRT-PCR confirmation of the expression of key genes-of-interest from RNA-seq analysis in: (**A**) MRSA 11/11, NaHCO_3_-responsive strain; and (**B**) COL, non-responsive strain. Expression was determined in log phase MRSA 11/11 and COL strains, each grown in the presence of ½ x MIC OXA. For each gene, expression is normalized to that observed for the indicated strain grown in CA-MHB Tris+2% NaCl + ½ × MIC OXA (CA-MHB Tris + 2% NaCl + ½ × MIC OXA expression set at 1.0). Statistics were calculated by Student’s *t*-test (*** *P* < 0.001, **** *P* < 0.0001).

The qRT-PCR validation of specific genes identified by RNA-seq revealed that *sceD* expression was strongly repressed by NaHCO_3_ in MRSA 11/11, while being substantially upregulated in the non-responsive strain COL (Figure 3); this finding further supports the notion that altered *sce*D expression may be of key importance to the NaHCO_3_-responsive phenotype. The qRT-PCR also validated the NaHCO_3_-mediated repression of *pbp2*, *atl*, and *fnbA* in responsive strain MRSA 11/11 as seen by RNA-seq, while NaHCO_3_ stimulated increased expression of these genes in COL (Figure 3).

### 3.3. Limitations of Study

This study was limited by only using a single NaHCO_3_-responsive/non-responsive strain pair for RNA-seq analyses. A larger number of strains exhibiting each phenotype would increase the likelihood of identifying a more consensus cadre of differentially expressed genes involved in the NaHCO_3_-responsiveness phenotype. Further, only one β-lactam (OXA) was tested for its impact on gene expression in the presence of NaHCO_3_. Our previous work has demonstrated that NaHCO_3_-induced sensitization to cefazolin (CFZ) is much more frequent among MRSA strains than sensitization to OXA [16]. This implies that CFZ and OXA may be impacting somewhat different genetic targets in NaHCO_3_-responsive strains, which could be causing the increased frequency of sensitization to CFZ vs. OXA. Additionally, only one concentration of NaHCO_3_ (44 mM) was examined for its impact on gene expression. Investigating the impact of NaHCO_3_ at blood concentrations (~25 mM NaHCO_3_) may reveal genes that are particularly important to NaHCO_3_-β-lactam-induced changes in expression within that specific host microenvironment.

Although KEGG analysis was carried out, the relatively small number of differentially expressed genes in MRSA 11/11 limited the number that could be functionally categorized to a selected, definable metabolic or biosynthetic pathway. The relative lack of descriptive KEGG pathways to ascribe to the differentially transcribed genes in these analyses underscores the need for separate functional characterizations of those genes we have identified by RNA-seq. Furthermore, additional investigations with a larger collection of NaHCO_3_-responsive strains may help in determining specific, consensus KEGG pathways associated with the responsive phenotype.

## 4. Conclusions

NaHCO_3_ (especially in combination with OXA) impacts a variety of genes specifically and differentially expressed in the NaHCO_3_-responsive strain MRSA 11/11 relating to: cell wall synthesis, turn-over and division; and cell surface-associated genes regulated by the *sigB-sarA*-*agr* regulatory axis. These analyses point to a mechanism of β-lactam sensitization by NaHCO_3_ that involves global impacts on the cell surface (where β-lactams initially bind), as well as altered expression of genes required to synthesize the cell wall and maintain its homeostasis. Moreover, further work is needed to validate and quantify the specific impact of those genes identified herein on the NaHCO_3_-responsive phenotype, using strategic gene knockouts. Finally, future studies will be designed to look at the differential impacts of NaHCO_3_ upon protein-level expression profiles in NaHCO_3_-responsive vs. non-responsive MRSA.

## Figures and Tables

**Table 1:** Overview of gene functional classes altered ≥ 2-fold in two comparison groups, (Group 1) CA-MHB Tris vs. CA-MHB Tris + NaHCO_3_, and (Group 2) CA-MHB Tris + 2% NaCl + OXA vs. CA-MHB Tris + NaHCO_3_ + 2% NaCl + OXA. Genes/transcriptional units indicated in this tables are those altered by NaHCO_3_ exposure alone (Group 1, *n* = 11), NaHCO_3_ and OXA co-exposure (Group 2, *n* = 57), or common to both groups (Group 1 and 2, *n* = 10) for a total number of functionally identifiable genes/transcriptional units of 78. "Increase" and "decrease" refer to expression being increased or decreased by exposure to NaHCO_3_ compared to media without NaHCO_3_ in each comparison group.

Classifications of Gene Functions	Number of Genes/Transcriptional Units Per Category	Number Increased	Number Decreased
Virulence	12	2	10
Cell Wall Synthesis	10	1	9
Membrane	19	4	15
Metabolism	22	12	10
Transcription	5	1	4
Translation	6	5	1
Transposition	2	2	0
Stress Response	2	1	1
Totals:	78	28	50

**Table 2:** Genes of interest selectively altered in NaHCO_3_-responsive strain MRSA 11/11 (log_2_ fold change ^A^).

Gene Name	Locus Tag	+NaHCO_3_ (no OXA)	+NaHCO_3_ with OXA ^B^	Function	−2	0	2
*clfA*	SAUSA300_0772	−2.18	−2.97	virulence			
*clfB*	SAUSA300_2565	1.29	1.11				
*agr*	SAUSA300_1989	−3.21	−5.37				
*sdrH*	SAUSA300_1985	−2.44	0.00				
*fnbA*	SAUSA300_2441	0.00	−1.84				
*fnbB*	SAUSA300_2440	0.00	−1.47				
*atl*	SAUSA300_0955	−1.65	−2.93	autolysins			
*sceD*	SAUSA300_2051	−6.53	−4.41				
*isaA*	SAUSA300_2506	0.00	−2.12				
*fmtA*	SAUSA300_0959	0.00	−1.32	peptidoglycan/cell wall synthesis			
*ddh*	SAUSA300_2463	0.00	−4.00				
*pbp2*	SAUSA300_1341	0.00	−1.81				
*bccT*	SAUSA300_2549	−2.45	−2.42	osmotic stress			
*usp*	SAUSA300_0067	−1.30	−2.28	stress response			

^A^ Fold change comparisons are made by comparing the gene expression for the indicated strain and condition (± OXA) exposed to NaHCO_3_ to the same strain/condition without NaHCO_3_ exposure. Negative values indicate that exposure to NaHCO_3_ for the given strain/condition decreases expression of the indicated gene, whereas positive values indicate that NaHCO_3_ increases expression of the indicated gene. ^B^ OXA concentration equal to 1/2 × MIC in CA-MHB Tris ± NaHCO3 for the indicated strain.

**Table 3:** Genes of interest altered in both NaHCO_3_-responsive and non-responsive strains (log_2_ fold change ^A^).

Gene Name	Locus Tag	MRSA 11/11 +NaHCO_3_ (no OXA)	COL +NaHCO_3_ (no OXA)	MRSA +NaHCO_3_ with OXA ^B^	COL +NaHCO_3_ with OXA ^B^	Function
*cap8*	SAUSA300_0152	−3.05	2.36	−4.45	0.00	virulence
*clpL*	SAUSA300_2486	0.00	0.00	−1.44	−1.46	
*sasD*	SAUSA300_0136	0.00	0.90	2.11	−1.82	
*aaa*	SAUSA300_0438	0.00	−1.14	−2.20	0.00	
*vraX*	SAUSA300_RS03005	0.00	0.00	−4.45	1.88	
*kdpABCDF*	SAUSA300_2032	0.00	0.90	−2.07	1.79	osmotic stress
*betAB*	SAUSA300_2545	−4.44	−1.38	−5.12	−4.28	
*icaR*	SAUSA300_2599	0.00	1.87	1.62	0.00	transcriptional regulator
*rsp*	SAUSA300_2326	−1.60	−1.02	0.00	0.00	

^A^ Fold change comparisons are made by comparing the gene expression for the indicated strain and condition (± OXA) exposed to NaHCO_3_ to the same strain/condition without NaHCO_3_ exposure. Negative values indicate that exposure to NaHCO_3_ for the given strain/condition decreases expression of the indicated gene, whereas positive values indicate that NaHCO_3_ increases expression of the indicated gene. ^B^ OXA concentration equal to 1/2 × MIC in CA-MHB Tris ± NaHCO3 for the indicated strain.

## Data Availability

The data presented in this study are openly available in the NCBI Sequence Read Archive (SRA) as indicated in the manuscript materials and methods.

## Supplementary Materials

The following are available online at https://www.mdpi.com/article/10.3390/genes12111650/s1, Table S1: qRT-PCR primers, Table S2: (A) Genes altered ≥ 2-fold by CA-MHB 100 mM Tris + 44 mM NaHCO_3_ exposure (without oxacillin) compared to CA-MHB 100 mM Tris alone (without oxacillin) specifically in NaHCO_3_-responsive strain MRSA 11/11, (B) Genes altered ≥ 2-fold by CA-MHB 100 mM Tris + 44 mM NaHCO_3_ with oxacillin exposure compared to CA-MHB 100 mM Tris alone with oxacillin exposure specifically in NaHCO_3_-responsive strain MRSA 11/11, (C) Genes altered ≥ 2-fold by CA-MHB 100 mM Tris + 44 mM NaHCO_3_ exposure (± oxacillin) compared to CA-MHB 100 mM Tris alone (± oxacillin) specifically in NaHCO_3_-responsive strain MRSA 11/11.
